# Combined Scleral Flap with Donor Scleral Patch Graft for Anterior Tube Placement in Glaucoma Drainage Device Surgery

**DOI:** 10.1155/2016/2124581

**Published:** 2016-09-25

**Authors:** Jea H. Yu, Chuck Nguyen, Esmeralda Gallemore, Ron P. Gallemore

**Affiliations:** ^1^Clinical Research, Retina Macula Institute, Torrance, CA, USA; ^2^University of California, Los Angeles, Los Angeles, CA, USA

## Abstract

*Purpose*. To report a new technique for anterior placement of tubes for glaucoma drainage devices to reduce the risk of tube erosions.* Methods*. Retrospective review of select cases of Ahmed Valve surgery combined with the novel method of a limbal-based scleral flap covered by a scleral patch graft to cover the tube at the entrance through the limbus. Intraoperative and postoperative illustrations are shown to highlight the method of tube placement.* Results*. In this retrospective case series, 3 patients are presented illustrating the technique. Two had neovascular glaucoma and one had primary open-angle glaucoma (POAG). On average, intraocular pressure was reduced from 39 ± 14 mmHg to 15 ± 2 mmHg and the number of glaucoma medications was reduced from 4 ± 1 to 0. Preoperative and most recent visual acuities were hand-motion (HM) and HM, 20/60 and 20/50, and 20/70 and 20/30, respectively.* Conclusion*. The combination of a limbal-based scleral flap with scleral patch graft to cover the tube with glaucoma drainage devices may be an effective means to reduce erosion and protect against endophthalmitis.

## 1. Introduction

Glaucoma Drainage Devices (GDD) (e.g., Ahmed, Baerveldt) are utilized in the management of complex cases of glaucoma, particularly in neovascular and uveitic and other intractable forms [1]. They provide an attractive alternative to trabeculectomy surgery, particularly in younger patients, with a lower risk of endophthalmitis [2, 3]. The TVT study demonstrated higher long-term success for tubes over trabeculectomy, although comparable rates of IOP control were achieved [4]. One disadvantage of GDD is erosion of the tube, particularly when placed through the limbus and into the anterior chamber (AC). Even with the placement of a patch graft, erosion rates of approximately 8% are reported [5] with an associated incidence of endophthalmitis as high as 2% of patients undergoing GDD surgery [6]. One alternative to this approach is posterior placement of the tube—into the pars plana [7]. This may reduce rates of erosion but not all patients are good candidates for posterior placement and proper posterior placement may require assistance of a retinal surgeon to clear the vitreous and prevent clogging of the tube. Here we report a novel technique for anterior placement of the Seton valve tube utilizing a scleral tunnel combined with a scleral patch graft. No cases of erosion or dellen formation were reported in our series.

## 2. Methods

**Figure 1:**
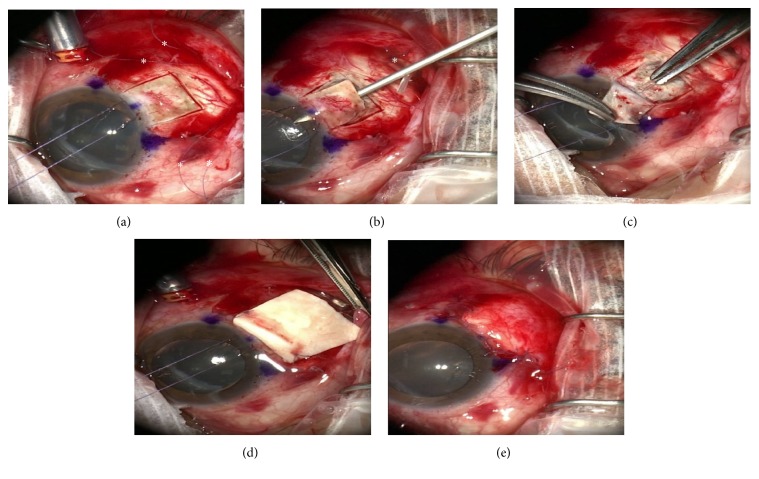
(a) Outline of scleral flap made with crescent blade. Note: 7-0 vicryl limbal retraction suture and marking of 9:00′, 10:30′, and 12:00′ positions (*∗*). (b) Ahmed Valve sutures in place (*∗*) and AC entered and formed with viscoelastic on a 23-gauge needle. (c) Tube cut and inserted into AC, beneath the flap. (d) Human scleral patch graft fixed in place 1-2 mm behind limbus 7-0 vicryl suture. (e) Conjunctiva closed with running locking 7-0 vicryl suture.

We report a retrospective case series of patients undergoing Ahmed Valve placement into the AC combined with vitreoretinal surgery, when indicated, utilizing a scleral tunnel technique.  Figure 1 illustrates an example case. All patients underwent retrobulbar anesthesia and after prompt preparation and draping, a 23-gauge infusion cannula was placed in the inferotemporal quadrant 3-4 mm posterior to the limbus. The infusion was attached to the cannula and directly visualized before turning on.

**Figure 2:**
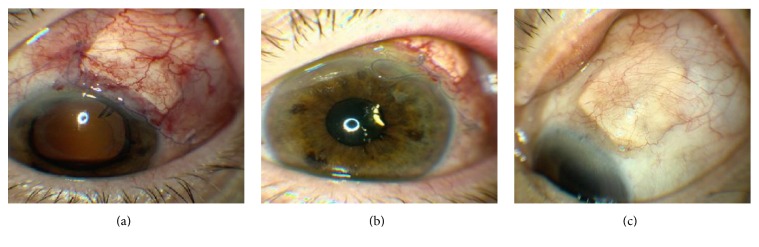
(a) 1 day s/p surgery. (b) 22 days s/p surgery. (c) 90 days s/p surgery.

Intraocular pressure (IOP) was set at 10–20 mmHg to protect the optic nerve from fluctuations of IOP during the procedure. A conjunctival peritomy was created in the superotemporal quadrant. The limbus was marked at 9:00 and 12:00 for the right eye and 12:00 and 3:00 for the left eye and at the midpoint between these two markings. A 7-0 vicryl stay suture was placed through the limbus at approximately 50% corneal depth and the eye rotated inferonasally. Westcott's scissors were used to bluntly dissect beneath the subtenons posteriorly and then the lateral and superior rectus muscles were localized with muscle hooks, whereafter two 7-0 vicryl sutures were preplaced for the Ahmed Valve between the two muscles, 9 mm posterior to the limbus and 5 mm apart. The limbal marking points were also used to help align the valve which was then positioned and sutured in place. We believe precise placement of the valve is critical to minimize strabismus related complications. A 5 × 5 mm limbal-based scleral flap was then created using a bent crescent blade (Figure 1(a)). The retraction suture was released and any vitrectomy procedures were then performed at this point. When placing the tube in the anterior chamber, care is taken to prevent both iris and/or corneal touch by placing a bent 23-gauge needle attached to a viscoelastic-like ProVisc through the surgical limbus parallel to the iris plane (Figure 1(b)) and maintaining the AC with a small amount of viscoelastic. The goal is to have the AC volume normalized—countering any aqueous loss that resulted from entering the chamber. The tube was cut at a 30-degree angle for ease of placement through the ostomy created by the 23-gauge needle (Figure 1(c)). A scleral patch or a piece of tutoplast was then sutured in place over the scleral flap (Figure 1(d)) and the conjunctiva was closed with a running locking 7-0 vicryl suture (Figure 1(e)). Also, we have not found it necessary to place anchoring sutures around the tube or scleral flap, perhaps due to the better compression and fixation of the grunge created by the triple layer coverage—scleral flap, donor scleral patch graft, and the conjunctiva. Examples of postoperative appearances are shown in Figure 2.

## 3. Results

### 3.1. Case  1

A 65-year-old Hispanic male with a history of proliferative diabetic retinopathy (PDR) and hypercholesterolemia developed a central retinal artery occlusion followed by neovascular glaucoma. IOP was elevated at 47 mmHg despite maximal medical management including dorzolamide 2% (Azopt Alcon, Fort Worth, TX), brimonidine 0.2%, timolol 0.5%, and travoprost ophthalmic solution 0.0004 (Travatan Z, Alcon, Fort Worth, TX). Pre-op visual acuity (VA) was hand-motion (HM). Ahmed Valve surgery with anterior placement of the tube beneath a limbal-based scleral flap, as described in the methods section, was performed. Infusion was attached to a pars plana cannula placed in the inferotemporal quadrant and was inserted using a 23-gauge trocar cannula system (Alcon Pharmaceuticals, Irvine, CA) with the IOP set at 20 mmHg. Pars plana vitrectomy with pan retinal photocoagulation (PRP) was performed for the treatment of PDR. A recessed scleral patch graft was placed 1 mm posterior to the limbus over the scleral flap. The sclerotomy sites were placed in the inferotemporal, superotemporal (beneath the conjunctival peritomy), and superonasal quadrant, 3.5 mm posterior to the limbus. On post-op day (POD) 1 the IOP was reduced to 11 mmHg while off all antiglaucoma medications and his VA was hand-motion (HM). On his most recent follow-up (post-op week 34) his IOP was 17 mmHg while off all antiglaucoma medications and his VA was HM.

### 3.2. Case  2

**Figure 3:**
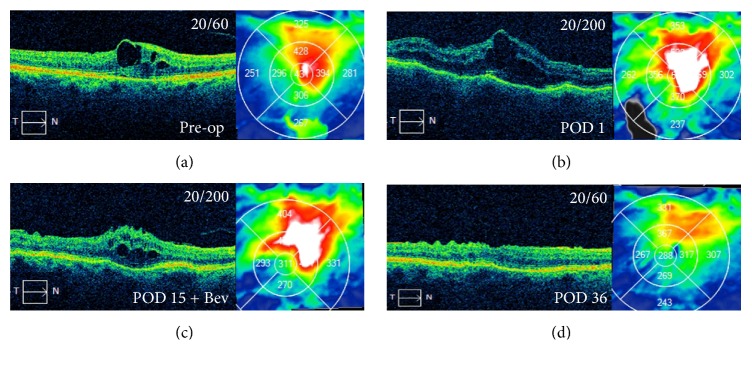
(a) Patient with a CRVO develops recalcitrant macular edema associated with the use of travoprost. Also she is struggling to achieve her target IOP despite maximal medical management and elects to proceed with Ahmed Valve surgery. Pre-op VA is 20/60. (b) POD 1, IOP reduced to 0 mmHg off all antiglaucoma medications but placed on topical steroid difluprednate (Durezol, Alcon) at QID for the postoperative edema. POD 1 VA is 20/200. (c) POD 15 VA is 20/200 and, given the residual edema, intravitreal bevacizumab injection is given. (d) POD 36 VA improves to 20/60; IOP stabilizes at 14 mmHg while off all antiglaucoma medications and edema starts to resolve.

An 81-year-old Caucasian female with a history of macular edema secondary to central retinal vein occlusion (CRVO) developed recalcitrant macular edema. She was taking topical brimonidine 0.2%, dorzolamide 2%, and travoprost ophthalmic solution 0.004% (Travatan Z, Alcon, Fort Worth, TX) for treatment of primary open-angle glaucoma. The travoprost use was associated with inflammation and recalcitrant macular edema (Figure 3(a)). She was also struggling to achieve her target IOP, despite maximal, tolerated, medical management. Her visual fields showed progressive arcuate defects and nasal step and OCT studies showed a correlated, progressive nerve fiber layer loss. For these reasons, she was offered and elected to proceed with Ahmed Valve surgery with anterior placement of the tube beneath a limbal-based scleral flap, as described in the methods section. Infusion was attached to a pars plana cannula placed in the inferotemporal quadrant and the IOP was set at 20 mmHg. A recessed scleral patch graft was placed 1 mm posterior to the limbus over the scleral flap. IOP measured 18 mmHg and VA was 20/60 at the last preoperative visit, 1 week prior to surgery. On POD 1, IOP was reduced to 0 mmHg and she was started on atropine 1% bid to use for only one week and neomycin-polymyxin-dexamethasone 3.5 mg/mL-10,000 unit/mL-0.1% qid and the topical steroid difluprednate (Durezol, Alcon) was continued at qid; her VA was 20/200 (Figure 3(b)). At POD 15, she had residual macular edema and received a bevacizumab injection (Figure 3(c)). At POD 36, her vision was 20/60, the IOP had stabilized at 14 mmHg, and the macular edema has improved as shown (Figure 3(d)). At her most recent follow-up (post-op week 38) her IOP was 16 mmHg while off all antiglaucoma medications and her VA was 20/50.

### 3.3. Case  3

A 49-year-old, Hispanic male with a history of macular edema secondary to central retinal vein occlusion developed neovascular glaucoma with a maximum IOP reading of 48 mmHg with a cup to disc ratio of 0.2. He was treated with a bevacizumab injection and was started on maximal, medical glaucoma management: brimonidine 0.2%, dorzolamide 2%, timolol 0.5%, bimatoprost ophthalmic solution 0.03% (Lumigan, Allergan, Irvine, CA), and Acetazolamide 250 mg tablets, reducing his IOP to 15 mmHg. After two more bevacizumab injections, his IOP increased to 25 mmHg while on the same regimen. He elected to undergo Ahmed Valve placement with limbal-based scleral flap for tube placement in the AC with a scleral patch graft. A pars plana maintainer was utilized during the procedure to keep the IOP at 20 mmHg. Pars plana vitrectomy with PRP was also performed for treatment of ischemic peripheral retina. On POD 1, the patient's IOP was well-controlled at 6 mmHg and his VA was count fingers at 4 feet while off all antiglaucoma medications. At his most recent follow-up (post-op week 28) his IOP was 22 mmHg, while off all antiglaucoma medications and his VA was 20/30.

## 4. Discussion

Glaucoma drainage devices (GDD) are effective for the management of complex cases of glaucoma refractory to medical management. Traditionally, the tube of the drainage device is placed into the AC through the limbus. The tube is then covered with a scleral or other patch graft overlapping the limbus corneal junction. Alternatively, a scleral flap may be used. By combining the two techniques, complications may be reduced. The scleral flap allows more posterior and deeper entry into the AC, reducing the risk of erosion at the limbus as well as the risk of dellen formation at the limbus. The overlying patch provides further protection from erosion of the entire tube and may be recessed a millimeter from the limbus when combined with the scleral flap, reducing cosmetic issues of the patch at the limbus as well as the risk of dellen formation. One of the most dreaded complications of GDD surgery is erosion of the tube through the conjunctiva and rates of 2.5–7.1% have been reported [8]. Subsequent endophthalmitis can occur with loss of vision and even loss of the eye [9]. To reduce the risk of erosion, grafts are placed over the tube at the corneal limbal junction with a wide range of material utilized including donor human sclera, pericardium, corneal buttons, and fascia lata. Erosion still occurs despite the placement of these grafts. The cause of erosion appears to be focal compression of the conjunctiva by the elevated tube combined with drying of the tissues caused by the irregularity of the surface at the junction of the cornea and limbus. In order to create a smoother entry of the tube into the AC and minimize compression of the conjunctiva by the tube, we have utilized a scleral tunnel technique for placement of the tube into the AC. This is a simple technique requiring only minutes to perform and when combined with the overlying scleral patch graft has eliminated all cases of tube erosion with anterior placement of the tube in our practice. Scleral flaps alone have been found to have a higher rate or tube erosion than a scleral patch graft alone [10] and the rates of erosion with the patch graft are as noted above. While we have utilized this technique only with the placement of an Ahmed Valve, it could be used for any drainage device. We suggest the use of a scleral tunnel with scleral flap for the placement of all GDD that are inserted into the AC to reduce the chance of conjunctival erosion, dellen formation, and endophthalmitis and to enhance the cosmetic results.
